# Development and assessment of a loop ligation simulator for laparoscopic appendectomy

**DOI:** 10.1007/s00383-024-05664-6

**Published:** 2024-03-21

**Authors:** Sabine Zundel, Noemi Singer, Lena Florinett, Jonathan Aichner, Tobias Jhala, Philipp Szavay

**Affiliations:** 1 Department of Pediatric Surgery, Children’s Hospital Lucerne, 6000 Lucerne 16, Switzerland; 2 University of Lucerne, Lucerne, Switzerland

**Keywords:** Loop ligation, Appendix, Appendectomy, Laparoscopy

## Abstract

**Objective:**

Loop ligation of the appendix is a challenging surgical skill and well suited to be trained in a simulator. We aimed to develop an affordable and easy-to-build simulator and test its training effect.

**Design and participants:**

Different materials were tested, and the best training modality was identified by researching the literature. The developed simulator training was tested on 20 surgical novices.

**Results:**

A video was produced including an instruction on how to build the simulator and a step-by-step tuition on how to ligate the appendix. The Peyton approach was utilized to guide learners. Training with the simulator leads to reliable skill acquisition. All participants improved significantly in completing the task successfully during the structured learning.

**Conclusion:**

We succeeded in developing a simulator for loop ligation of the appendix during laparoscopic appendectomy. Participants significantly improve in handling the loops. The transferability of the skill learned during simulation to the operating room will be subject of a follow-up study.

## Introduction

Surgical education is in the process of changing and surgical skills are increasingly learned and practiced on models and in simulators [1]. Laparoscopic appendectomy is a very common procedure [2], and it is a procedure typically taught to surgical residents early in their carrier. The procedural steps are standardized and therefore well suited to be practiced in a simulator.

Numerous devices can be used to ligate the appendix. Endoloops or endostaplers are most commonly applied [3, 4]. Technically, the use of endoloops is more challenging [5], but they are considerably less expensive and produce a fraction of the waste.

While the fundamentals of laparoscopic surgery test includes an endoloop simulation, no medium fidelity model exists to practice the skill of loop ligation of the appendix for laparoscopic appendectomy.

With this study, we aim to develop and test a simulator to teach and practice the ligation of the appendix with endoloops. Target groups for this simulator training are surgical trainees at the beginning of their career as well as experienced surgeons who have little experience with endoloops for laparoscopic appendectomy. The manuscript addresses the ACGME Core Competencies Patient Care, Practice-Based Learning, and Improvement.

## Materials and methods

### Development of the simulator hardware

Requirements for the appendix model were identified: inexpensive, easily available material, and a haptic as similar to an appendix, as possible. Different materials were tested for both, cover and filling, including plastic bags, surgical drains, gloves, ultrasound probe covers, water, gel, baby food, and sponges. Each combination was tested by the authors SZ, LF, and TJ.

**Fig. 1:**
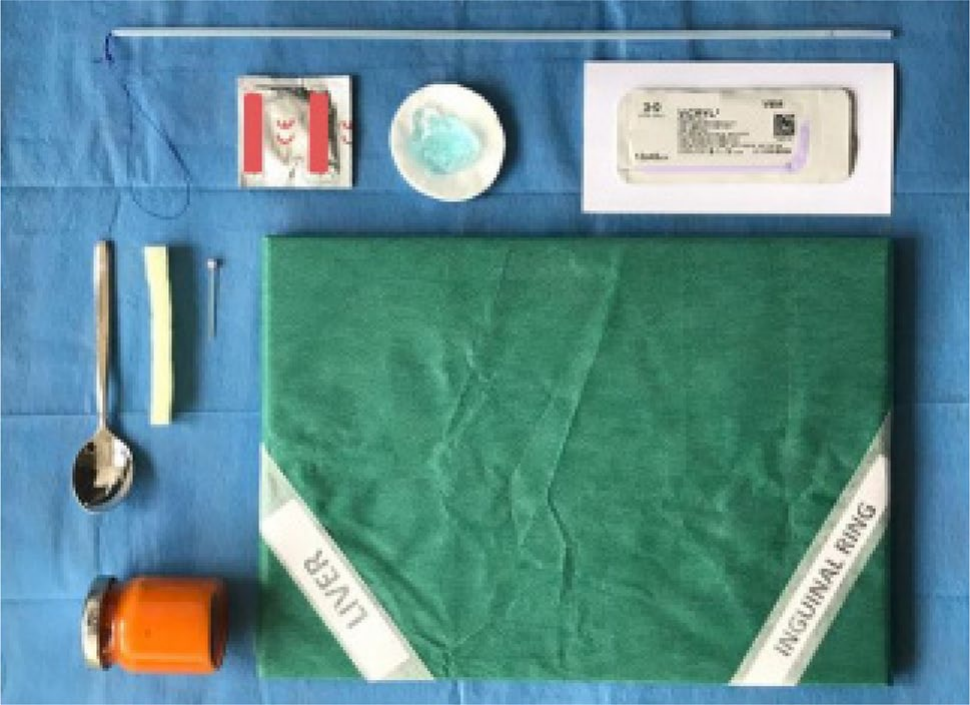
Material necessary to build the simulator

The material with the best haptic functions was an ultrasound probe cover (or condom) filled with a piece of sponge and mashed carrots, and lubricated with ultrasound gel. The material necessary to build the model is shown in Fig. 1.

### Development of an educational strategy and teaching video

**Fig. 2:**
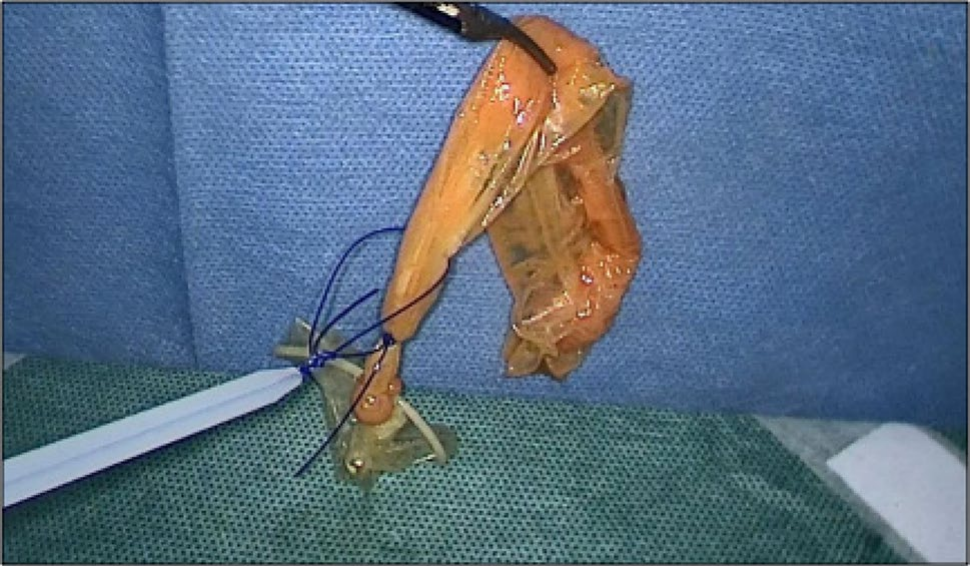
Appendix model in the pelvic trainer

PubMed and Google Scholar were searched for a suitable educational strategy. As a well-known and well-validated tool, “Peyton’s four-step approach to skill acquisition” [6] was chosen. To allow for teacher-independent learning, a teaching video was created and published [7]. The video guides the learner through the four steps of Peyton’s approach and shows and verbalizes the use of the instrument and manipulation of the appendix in detail. One picture from the video is displayed in Fig. 2.

### Early experience with simulator application

To test the expediency of the simulator, 20 medical students from the Joint Medical Master of Zurich and Lucerne participated in this study. All participants were surgical novices, and all agreed to have the data relevant to this study published. The study was not part of their standard curriculum, the participation entirely voluntary and not compensated. Data storage was done anonymously, and care was taken that neither participation nor non-participation or individual performance influenced the students’ grading in any courses of the current semester.

As a first step, the participants watched the teaching video and performed Peyton’s four steps. Afterward, each participant ligated four appendices with three loops each (12 loops total). This training was repeated on day 2 and day 4. The camera was driven by the author NS during all practicing episodes to avoid a learning effect by watching others perform. Time until correct placement of each loop was measured. For the statistical analysis of the measurements, Microsoft Excel™ and SPSS™ were used. Friedmann test was utilized to test for statistical significance. As multiple pairwise tests were performed, we used the Bonferroni correction to reduce the chances of type 1 errors. *p* < 0.05 was defined as statistically significant.

## Results

All participants understood the task and were able to perform it after watching the teaching video. No additional explanation or advice was necessary. Two participants were not able to participate on day 4 and their data was therefore excluded from further analysis.

Mean time to place the first loop (App 1.1) was 65.22 s, median = 50.5 s with the fastest result being 38 s (minimum) and the longest time taken being 122 s (maximum). After placing a total of 36 loops spaced out over three days, mean time for the last loop was 12.94 s, median = 12.5 s. Minimum time needed to place the last (36th) loop was 6 s, maximum being 26 s.

As obvious from the descriptive data, all participants became much faster with training. Even without statistical testing, these results demonstrate a clinically relevant skill acquisition with training. For completeness, the significance was calculated and proved to be *p* < 0.001.

**Fig. 3:**
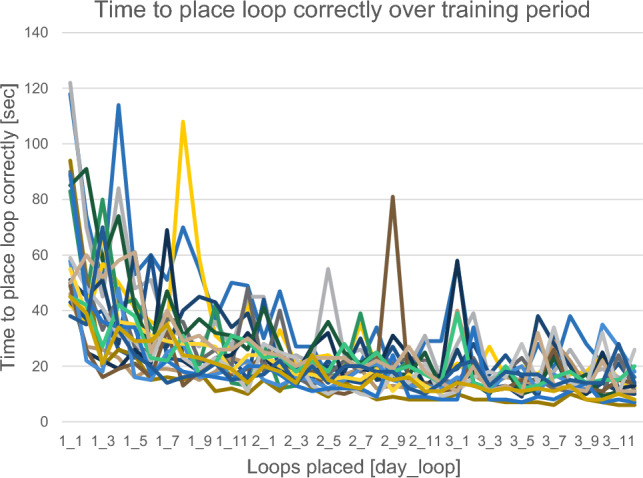
Time needed to place loop correctly over training period

A prediction on future performance from time needed at the beginning of the training did not prove to be possible. Initially, slower participants were not slower in the last training than those that were fast at the beginning and vice versa. Improvement during training is displayed in Fig. 3.

To further evaluate the effect of training, we evaluated the first loop placed on each day to the last loop placed on that day (training effect per day). On day 1, the improvement was not statistically significant with adjusted *p* = 0.075. Therefore, when first confronted with the task on day 1, the participants did not improve significantly with 12 repetitions.

This changed on day 2 and day 3 with adjusted *p* = 0.015 on day 2 and *p* = 0.017 on day 3. Therefore, repetitive training with resting periods in between proved effective. The statistical data are displayed in Table 1.

**Table 1:** Comparison of Median improvement per day

Day_loop	Test statistic	Std. Error	Std. test statistic	Sig	Adj. Sig.^a^
1_12 1_1	1.750	0.624	2.806	0.005	0.075
2_12 2_1	2.056	0.624	3.296	< 0.001	0.015
3_12 3_1	2.028	0.624	3.252	0.001	0.017

Asymptotic significances (2-sided tests) are displayed. The significance level is 0.050

^a^Significance values have been adjusted by the Bonferroni correction for multiple tests

Additionally, the medians of each day were compared, testing the improvement of each training episode. We found a statistical significance improvement between day 1 and day 2 (*p* = 0.003) but none between day 2 and day 3 (*p* = 0.059) even though there was a day of no practice between day 2 and day 3.

## Discussion

With the demonstrated model, simulating the ligation of the appendix with endoloops worked well and participants were able to improve performing the task with deliberate practice. We succeeded in developing a model to learn and train the specific skill of loop ligation for laparoscopic appendectomy.

### Expedience of the simulator

Breaking down the whole laparoscopic appendectomy into individual tasks does make sense from a medical education standpoint: Traditional models of skill acquisition and expertise distinguish different stages of learning: novices apply rules and perform procedures step-by-step [8]. Training with a step-by-step directory of how to handle the appendix and place the loop is an exercise customized specifically to the needs of the beginner. With the detailed instructions in the teaching video, the learners were well prepared for starting their training. Applying a hands-on model to train a practical skill is a prerequisite: simulators are known to develop hand–eye coordination and dexterity [9]. Loop placement is particularly suitable for simulator training as the task is remarkably consistent once the appendix has been freed from adhesions and the vessels been divided.

### Educational strategy

Handling the loop and pusher is not as intuitive as the use of other surgical instruments. Some loop ligation experts might have excelled in performing the task but might not be able to or care to verbalize their strategy. Even if they are motivated to be good teachers, data suggest that “their ideas about good teach is rooted in their own educational experiences and memories which are context-based and generally rather unstructured” [10]. Therefore, a teacher’s approach might not be helpful for the inexperienced resident. The presented video offers one way of loop placement and disassembles the task into easily conceivable steps, thus allowing the learner to grasp the task independent of a teacher’s skill and without trying the patience of his or her tutor.

The Peyton approach has been shown to be well suited to learn laparoscopic skills: Romero et al. for example compared laparoscopic suturing and knot tying taught by either demonstration or the Peyton approach and found the Peyton group significantly outperforming their peers [11]. These evident findings are backed up by a conceptual framework: in the cognitive process of psychomotor skill acquisition, five components can be identified: Attention, Perception, Concept formation, Memory, and Learning [12]. The Peyton approach addresses each component.

At the end of the instructional video, established training strategies are explained to allow for a maximum benefit. Particularly, the concept of mastery learning [13] with accompanying feedback and corrective measures by a tutor is essential to optimize the learning experience.

### Opportunity for assessment

Entrustable professional activities (EPAs) are becoming introduced in many surgical residency programs [14]. The presented simulator offers an opportunity to evaluate a trainee’s laparoscopic skill objectively and in a standardized way outside the operating room. An assessment on the simulator might therefore be well suited to be incorporated in an EPA to judge residents’ competency.

A transfer of the acquired skill to the operating room is likely as there is good evidence that procedural simulation improves operational performance in clinical settings [15]. We are currently implementing the simulator training in the residents’ curriculum at our institution and will assess this transferability by measuring performance.

During the expedience study, time was used to assess participants’ performance. In a previous study, analyzing essential laparoscopic tasks, we were able to demonstrate that time is an adequate measurement if the result of a task is clearly defined [16]. If a loop was not placed correctly or the string cut before the knot was secure, an additional loop had to be placed which took time.

One shortcoming of the model is that the material is much more durable than an infected appendix. The necessary care in handling the appendix cannot be experienced during the simulation and too rough a handling cannot be detected by measuring time. This further emphasizes the necessity of regular observation and feedback by tutors, as mentioned above.

### Application

There are many studies comparing different methods of stump closure during laparoscopic appendectomy [3, 4, 17, 18]. All methods lead to a sufficient closure and similar clinical outcomes. Therefore, the choice of the best technique needs to be defined by parameters other than patient outcome. Several studies have compared cost. A recently published review summarizes 25 studies and found the use of ligature, endoloops or endoclips cost-effective compared to endostaplers [19].

Although some authors with higher priced operating room time justified the use of staplers with the longer operative time needed if loops are placed [20, 21]. Miyano et al. wrote in 2011 “it will indeed be a sad day when cost alone dictates treatment, but should that day eventuate, then surgeons will need to retrain, accept that there is a learning curve for using unfamiliar techniques [4]. We do not believe this day has arrived yet, but with the help of our simulator, surgeons can become familiar with loops and transfer the learning curve out of the operating room.

No publication has analyzed the amount of waste produced by the larger endostaplers, but it is apparent that the amount of waste produced by endostaplers is significantly higher. The use of endoloops is therefore not only cost-effective but better for the environment.

To conclude, the application of this easy-to-use simulator allows for meaningful training and assessment during surgical training. By publishing our findings, we hope to further equip the toolbox of surgical teachers and thus improve surgical education.

## Conclusion

We succeeded in developing a teaching video and easy-to-build simulator for loop ligation. Training with the simulator leads to reliable skill acquisition. The transferability of the skill learned during simulation to the operating room will be subject of a follow-up study.

## Data Availability

No datasets were generated or analyzed during the current study.
